# Modulation of the Immune Environment in Glioblastoma by the Gut Microbiota

**DOI:** 10.3390/biomedicines12112429

**Published:** 2024-10-23

**Authors:** George B. H. Green, Alexis N. Cox-Holmes, Anna Claire E. Potier, Gillian H. Marlow, Braden C. McFarland

**Affiliations:** 1 Department of Cell, Developmental and Integrative Biology, Birmingham, AL 35294, USA; 2 Undergraduate Cancer Biology Program, Birmingham, AL 35294, USA

**Keywords:** microbiome, glioma, glioblastoma

## Abstract

Studies increasingly support the role of the gut microbiota in glioma development and treatment, although the exact mechanisms remain unclear. Research indicates that the gut microbiota can influence glioma progression, response to therapies, and the effectiveness of treatments like immunotherapy, with certain microbial compositions being linked to better outcomes. Additionally, the gut microbiota impacts the tumor microenvironment, affecting both tumor growth and the response to treatment. This review will explore glioma, the gut microbiota, and how their interaction shapes glioma development and therapy responses. Additionally, this review examines the influence of gut microbiota metabolites, such as short-chain fatty acids (SCFAs) and tryptophan, on glioma development and treatment. It also explores gut microbiome signaling via pattern recognition receptors, and the role of molecular mimicry between microbial and tumor antigens in glioblastoma, and if these interactions affect glioma development and treatment.

## 1. Introduction

The gut microbiota is comprised of bacteria, fungi, microalgae, and protozoa. The majority of the gut microbiota is comprised of bacteria, which inhabit the human gut, containing nearly 100 trillion members inside of the digestive system [1]. The human microbiota begins developing at the time of birth, and the composition is shaped via a plethora of factors such as mode of delivery, nutrition, and early environmental exposure. As individuals get older, the microbiota becomes more diverse until stabilization as an adult; however, chronic diseases such as obesity, diabetes, and cancer can disrupt the gut microbiota, which may result in exacerbating disease progression and weakening immune response [2]. These bacteria are essential for maintaining immune and metabolic homeostasis, as well as defending against pathogens. The immune system is strongly influenced by the gut microbiota with nearly 80% of immune cells being in attendance of the gut. The resident gut microbiota aids in the maturation of gut-associated lymphoid tissue (GALT) and maintains the barrier function via mucus and antimicrobial peptide production. Additionally, GALT can influence inflammatory cell phenotypes [3]. Over 15% of the cells present in the gut epithelium are T lymphocytes. Additionally, B lymphocytes, dendritic cells, and plasma cells are present in lymphoid tissues, which are in the colon lamina propia or Peyer’s patches [4]. Previous research has concluded that the gut microbiota is imperative for the function of immune cells, as well as overall metabolic health [5].

Gliomas are a group of tumors that originate from glial cells present in the brain and spinal cord, which is the most common primary brain tumor in the central nervous system [6].These tumors can vary in aggressiveness and prognosis, ranging from a low-grade pilocytic astrocytoma to highly malignant glioblastoma (GBM). It is estimated that each year in the United States, there are between 80,000 and 90,000 new cases of primary brain tumors, with about 25% of them being gliomas. The primary treatment approach is surgical resection, with the aim of removing as much of the tumor as safely as possible, depending on its grade and location. High-grade gliomas, such as GBM, present several complications that greatly affect patient outcomes and quality of life. The median survival rate for those with GBM is 15–16 months, and the 5-year survival rate is 5–10% [7]. The main challenges of treating GBM include (1) tumor heterogeneity, which enables the creation of resistant subpopulations; (2) tumor location, which is protected and complicates the delivery of therapies; and (3) significant local immunosuppression, which promotes immune evasion and reduces the effectiveness of new immunotherapies [7]. GBM is noted by the World Health Organization as a grade 4 diffuse glioma, which is an aggressive brain tumor [8,9]. Immunotherapy may hold promise in the treatment of GBM, but it has obstacles it needs to overcome due to the immunological environment of the central nervous system (CNS). The CNS location, as well as immunosuppressive mechanisms, make the tumors immunotherapy-resistant. While immunotherapy, such as immune checkpoint blockade (ICB), has been successful in treating other cancers, it has not yet demonstrated significant success in GBM [10]. ICB utilizes antibodies to target specific immune checkpoints, e.g., programmed cell death-1 (PD-1) and PD-ligand 1 (PD-L1) and cytotoxic T lymphocyte-associated protein 4 (CTLA-4) [11]. Recent evidence indicates an association of gut microbes with successful immunotherapy treatment. For example, mice with a diminished gut microbiota (via antibiotics and housing in a germ-free environment) resulted in a reduction in response to CTLA-4 blockade compared to mice with a gut microbiota [12,13,14].

**Figure 1:**
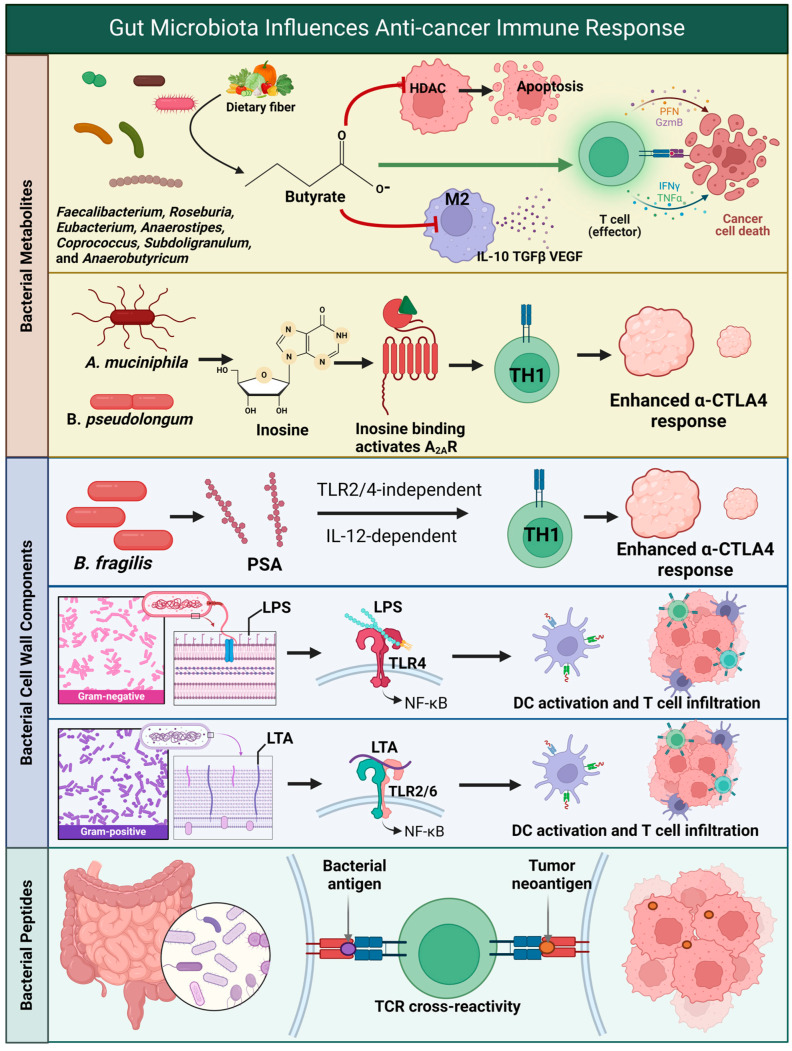
Summary of the relationship among the microbiome, metabolome, brain, and glioblastoma. Abbreviations: Histone deacetylase (HDAC), M2 Macrophage (M2), Interleukin-10 (IL-10), Transforming growth factor beta (TGFβ), Vascular endothelial growth factor (VEGF), Perforin (Prf), Granzyme B (GzmB), Interferon gamma (IFNγ), Tumor necrosis factor alpha (TNFα), *Akkermansia muciniphila* (*A. muciniphila*), *Bifidobacterium pseudolongum* (*B. pseudolongum*), Adenosine A2a receptor (A2AR), T helper 1 cell (TH1), Anti-cytotoxic T-lymphocyte-associated protein 4 (α-CTLA4), *Bacteroides fragilis* (*B. fragilis*), Polysaccharide A (PSA), Toll-like receptor 2 (TLR2), Toll-like receptor 4 (TLR4), Interleukin-12 (IL-12), Lipopolysaccharide (LPS), Nuclear factor-kappa B (NF-κB), Dendritic cell (DC), Lipoteichoic acid (LTA), and T cell receptor (TCR). Created in BioRender. Cox, A. (2024) BioRender.com/u44g091. Available online: https://app.biorender.com/citation/67165b1fe8646874611b6bae (accessed on 20 October 2024).

This review aims to address what is currently known about gliomas, the gut microbiota, and how the gut microbiota may affect the overall efficacy of immunotherapy. Lastly, we will address the clinical translational application of the gut microbiota [15] (Figure 1).

## 2. Glioma Tumors

Gliomas tumors account for 28% of all brain tumors and 80% of all malignant brain tumors with the most aggressive being GBM [16,17,18,19,20]. To diagnose and prepare a treatment regimen, conventional diagnostic tools are required such as biopsies, computed tomography (CT) scans, magnetic resonance imaging (MRI), and polyethylene terephthalate (PET) scans. The therapeutic options following the diagnosis depend on the grade and location of the tumor but often consist of surgical resection followed by chemotherapy and radiation. Surgery is often the first step to reduce cancer spread and decrease the side effects, but it is complex and not curative for advanced tumors such as GBM. In cases where a gross total resection is not a safe possibility for the patient, other avenues, like radiotherapy and chemotherapy, are considered. Radiotherapy utilizes large volumes of radiation targeted to specific affected regions. Typically, after surgery, patients will undergo roughly six weeks of radiation with each session increasing in volume if tolerated [21].

The other commonly used option is chemotherapy. However, chemotherapy can have detrimental effects on the body due to its aggressive nature, so it is not a recommended option for elderly individuals with a low functional status [22]. Temozolomide (TMZ) is currently considered the most effective chemotherapy drug for patients with advanced gliomas as this alkylating agent is one of the few cancer treatments that can cross the blood–brain barrier and enter the nucleus of the cell [23]. This commonly used drug is not beneficial to patients with an unmethylated O6-methylguanine-DNA methyltransferase (MGMT) gene promoter when used alone as it leads to drug resistance. MGMT encodes for an enzyme involved in DNA repair and protects cells from mutations; however, methylation of the MGMT promoter results in a decrease in MGMT expression, which results in a reduction in overall DNA repair, enabling cancer cells to become sensitive to chemotherapy. So, when the promoter is unmethylated, the cancer cells may develop resistance to chemotherapy [24]. Due to the invasive properties of these brain tumors, treatment options are limited and the prognosis for patients diagnosed with these malignant brain tumors is typically poor, as the average survival for a patient diagnosed with GBM is about 15 months [25].

In 2023, it was estimated that there were 24,810 new diagnoses and 18,990 deaths of CNS-related cancers [26]; therefore, thousands of people are diagnosed with gliomas, yet the majority of diagnosed adults do not have a cancer predisposition syndrome nor a family history of such diagnoses [27]. Additionally, males are more likely to develop aggressive brain tumors compared to females, with GBM incidences being higher in males [28]. Although these trends have been identified, the reasons for these differences remain unclear.

Gliomas often contain mutations that significantly affect tumor behavior and patient prognosis. Many gliomas, especially oligodendrogliomas, have mutations in isocitrate dehydrogenase 1 (IDH1) or isocitrate dehydrogenase 2 (IDH2), as well as deletions of chromosome arms 1p and 19q [29,30]. Over 80% of grades 2 and 3 gliomas carry IDH mutations, often IDH1 gain-of-function mutations, which prevent the formation of hydrogen bonds with the alpha and beta carboxyl sites in isocitrate and disrupt metabolic processes [31,32,33]. Gliomas with IDH mutations frequently show improved responses to treatment, so these mutations are a focus of targeted therapy research, including the newly FDA-approved vorasidenib for patients with IDH-mutated gliomas [34,35]. There are two main subtypes of IDH-mutated gliomas: one with 1p/19q codeletions and potentially telomerase reverse transcriptase (TERT) promoter mutations and another with mutations in ATRX and TP53. The 1p/19q codeletion subtype may reduce immune checkpoint gene expression, and ATRX mutations are believed to impact the tumor microenvironment, though the exact mechanisms remain unclear. GBM is the term designated for grade 4 with IDHwt (no mutation) [9]. Mutations in epidermal growth factor receptor (EGFR), the TERT promoter, and chromosomal abnormalities (such as gain of chromosome 7 and loss of chromosome 10) are common in more aggressive tumors like GBM [27]. TERT promoter mutations can lead to increased transcriptional and telomerase activities, increasing cancer cell viability and survival. Ongoing research aims to better understand these mutations to develop personalized therapies [36].

## 3. The Influence on Microbial Composition and Cancer

Since the NIH initiative of the Human Microbiome Project, microbiome research has continued to grow and how the gut microbial composition can impact overall wellbeing and medical treatments has become a popular focus of research [37]. With trillions of microorganisms found in the gut microbiota, there are several predominant ones. Firmicutes and Bacteroidetes comprise 90 percent of the gut microbiota, while the other 10 percent is comprised of Actinobacteria, Proteobacteria, Fusobacteria, and Verrucomicrobia [38].

Cancer has been largely thought to be a consequence of both genetic and environmental factors; however, the gut microbiota plays a role in cancer development and therapy [39]. Microbial composition may be directly oncogenic via systemic dysregulation and mucosal inflammation, and it may affect the efficacy of immunotherapy and drugs [35,37]. The process of carcinogenesis is closely tied to the immune system, which the gut microbiota supports. Previous studies utilizing a murine model have revealed that mice with disrupted gut microbiota had perturbed innate and adaptive immune system functions, which are important biomechanisms for the regulation and destruction of cancerous cells [40]. Cancer coupled with gut microbiota dysbiosis may result in a failure of the immune system to combat the tumor [41,42]. Lastly, patients with GBM are often prescribed trimethoprim–sulfamethoxazole and dexamethasone to prevent secondary infections and reduce edema, which can also affect the gut microbiota and may affect overall treatment and tumor progression [43,44].

The role of the gut microbiota is commonly associated with nutrient metabolism, drug and xenobiotic metabolism, maintaining the structural integrity of the mucosal barrier in the gut, immunomodulation, and protection against potential pathogens [45]. A byproduct of microbial nutrient metabolism is the production of short-chain fatty acids (SCFAs). The production of SCFAs occurs when bacteria ferment fiber consumed via the host and act as an energy source for the host. SCFAs present in the gut microbiota are predominately comprised of acetate, propionate, and butyrate [46]. Butyrate has regulatory effects on overall health, as well as anti-tumor effects. The anti-tumor effect of butyrate occurs due to modulation of immune responses, influence on tumor inflammatory microenvironment, inhibition of tumor proliferation, efficacy of immunotherapy, and maintenance of intestinal epithelial barrier function [47]. Propionate, while utilized as an energy source in gut cells, is transferred to the liver where it aids in the process of gluconeogenesis [48]. Additionally, propionate inhibits tumor growth via PPAR-γ signaling (peroxisome proliferator-activated receptor) [49]. Lastly, acetate may be utilized as a nutritional source for cancer cells, as acetyl-CoA conversion may be implicated in the growth of hepatocellular carcinoma, GBM, breast cancer and prostate cancer; however, the role of acetate in tumorigenesis is not fully understood as previous studies have shown acetate supplementation enhances the metabolic activity of T cells, boosting their effector functions and promoting increased proliferation [50,51].

In addition, members of the gut microbiota play a major role in the immune system, with studies suggesting they promote the production of cytokines, which may lead to a reduction in carcinogenesis in cells [52,53]. Previous data have shown that the gut microbiota influences the development of the immune system [54] and affects the progression of human diseases, particularly cancer [55]. The gut microbiota aids in the maintenance of intestinal balance, but disruptions can lead to inflammation and the release of inflammatory factors, influencing the tumor microenvironment (TME) and the regulation of immune cells [56]. Additionally, the gut microbiota can affect the efficacy of immunotherapy by modulating both innate and adaptive immune responses through the synthesis of metabolites and other regulatory mechanisms. Previous research has highlighted the interaction between microbial antigens and tumor antigens (observed across small-cell lung cancer, hepatocellular carcinoma, melanoma, and glioma) and how it plays a role in the anti-tumor effects of the gut microbiota [57,58]. As a result, microbial components may aid in the overall effectiveness of immunotherapy.

The future of cancer treatment is using targeted immunotherapy to directly attack the cancer instead of using broad treatments like chemotherapy. As new treatments have been developed, the role of the gut microbiota in treatment efficacy has gained attention. A 2015 study aimed to explore the effect of the gut microbiota composition on tumor growth and development in mice with melanoma. Mice with an increase in tumor-specific CD8+ T cells had a higher prevalence of *Bifidobacterium*. Researchers found that the administration of *Bifidobacterium* alone enabled tumor control which was comparable to anti-PD-L1 therapy and the combination of *Bifidobacteria* and anti PD-1 therapy had the greatest effect against tumor growth [14,59]. This study was groundbreaking because it supported that the gut microbiota played a more significant role in cancer regulation than previously thought. Research is now focused on pairing preferred gut microbiota with specific cancer treatments and examining the mechanisms of how these preferred gut microbiota achieve such beneficial outcomes regarding cancer growth and control [60].

## 4. The Effect of Microbial Composition on Glioma

The gut microbiota has been previously linked to the CNS [61] via the gut–brain axis; however, the mechanisms of how the microbiota interacts with glioma are unknown [61,62]. The human gut microbiota is associated with various CNS diseases, which include Alzheimer’s disease [63], Parkinson’s disease [64], and multiple sclerosis [65]. Therefore, the gut microbiota may play a role in the pathogenesis and pathophysiology of glioma. This may occur via the metabolic products synthesized via microbes which then affect the development of gliomas and the efficacy of therapies, as these metabolites affect the glioma environment and the immune response [66]. The gut microbiota maintains immune homeostasis in the brain via impacting the function of microglia, T cells, dendritic cells (DCs), macrophages, and other immune cells. Additionally, glioma tumorigenesis will affect immune homeostasis, as glioma cells dysregulate intracellular metabolites enabling them to proliferate quickly [18]. The brain has a distinct immune environment, which is linked to the gut microbiota and brain tumors via the gut–brain axis. This connection is protected by the blood–brain barrier (BBB), a specialized membrane made up of endothelial cells. The BBB controls which soluble substances, such as antibodies, metabolites, signaling molecules, and immune cells, enter the CNS [67].

Previous studies have indicated a potential causal association between the commensal gut microbiota and GBM. Wang et al. (2024) revealed that increased abundances of Ruminococcaceae are associated with having a lower risk of developing GBM [68]. Members of Ruminococcaceae synthesize the metabolite isoamylamine (IAA), which promotes microglia cells via the recruitment of the p53 transcript regulator to the S100A8 promoter region [69]. Additionally, members of the genera *Faecalibacterium*, *Roseburia*, *Eubacterium*, *Anaerostipes*, *Coprococcus*, *Subdoligranulum*, and *Anaerobutyricum* produce the majority of butyrate by metabolizing carbohydrates through the butyryl-CoA CoA-transferase pathway and the butyrate kinase terminal enzymes [70]. Butyrate is primarily known as an energy source for colonocytes and a beneficial metabolite for gut health; however, it also exhibits anti-tumor effects, such as reducing tumor necrosis factor (TNF) levels, which in turn may lead to decreased tumor growth. Lastly, butyrate functions as a histone deacetylase (HDAC) inhibitor, facilitating the acetylation of histones. This process allows for the reactivation of genes that play a role in tumor suppression, inhibiting cancer cell growth and promoting apoptosis [42,47].

The gut microbiota has the potential to exert anti-tumor effects, and this was seen in Wang et al. (2022) where they used an orthotopic mouse glioma model supplemented with *Bifidobacterium lactis* and *Lactobacillus plantarum*. This resulted in decreased tumor volume, increased survival time, and improved intestinal barrier. *L. plantarum* and *B. lactis* inhibited the PI3K/AKT pathway, which resulted in an overall decrease in Ki-67 (a marker used to indicate active cell proliferation in tumor cell populations) and N-cadherin (promotes cancer invasion, adhesion, metastasis, apoptosis, and angiogenesis) [71]. Additionally, other bacteria have shown anti-tumor or tumor-killing capability, such as *Bifidobacterium*, *Listeria*, *Salmonella*, *Escherichia coli*, and *Clostridium* [72]. The effect and mechanism are unknown in the model of glioma. Future research is focused on utilizing these anti-tumor effects via using bacteria as live tumor-targeting bacteria, which may be applied as a monotherapy or with other anti-cancer therapies [73].

Increasing evidence suggests a relationship between the gut microbiota and immunotherapy efficacy. Dees et al., 2021 utilized a novel humanized mouse microbiome and demonstrated that successful treatment of anti-PD-1 therapy is influenced via microbial composition in a murine model of glioma [59]. *Akkermansia municiphila* has previously been correlated with successful response to PD-1/PD-L1 therapy in patients with epithelial tumors [74]. Fecal microbiota transplants have been shown to reverse an unsuccessful response to PD-1/PD-L1 therapy in humans and mice [75,76,77]. *A. muciniphila* and *Bifidobacterium pseudolongum* may support successful immunotherapy via the inosine-A2AR signaling pathway [78]. Additionally, a preclinical study utilizing an anti-PD-L1 treatment saw a relationship between *Bifidobacterium* and anti-tumor T cell response [12]. Lastly, a higher alpha diversity with an increase in Ruminococcaceae and *Faecalibacterium* members has been linked to melanoma patients successfully responding to anti-PD-1 therapy [79]. ICB therapies have shown success in other cancers, as well as glioma preclinical models, but there has been little to no success in trials for patients with GBM [18]. Lastly, Meléndez-Vázquez et al. 2024 supported that the microbial composition was linked to successful oncolytic viral therapy, with higher abundances of *Bifidobacterium* being linked to a higher survival rate in a murine model of glioma [80].

CTLA-4 is a surface receptor on T cells that, when activated, inhibits T cell activity to regulate the immune response [81]. However, certain tumors can increase the expression of CTLA-4 and the associated ligands within the tumor microenvironment, enabling them to escape immune detection by suppressing T cell function. Previous data have revealed that an increased abundance of *Burkholderia cepacian*, *Faecalibacterium* members, and *Bacteroides fragilis* in patients prescribed CTLA-4-immunotherapy resulted in a stronger response effect, with overall fewer side effects associated with the therapy [82]. Additionally, germ-free mice administered CTLA-4 therapy failed to respond to treatment; however, the presence of a microbial composition enabled a successful response to CTLA-4 therapy [13,83]. *B. fragilis* was linked to an immunoregulatory function via the CTLA-4 pathway as it synthesized polysaccharide A, which may enhance IL-10 production, thereby increasing CTLA-4 expression and reducing inflammation [84].

## 5. Metabolites Produced via the Gut Microbiota and the Effect on Glioma

**Figure 2:**
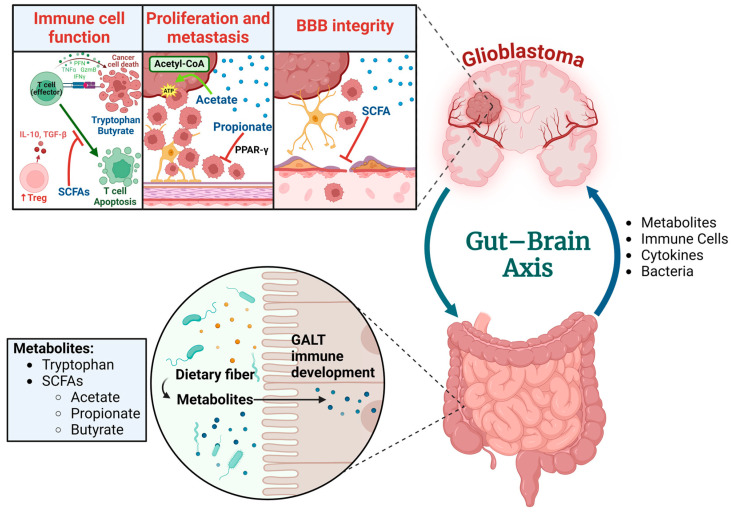
The interaction among metabolites, immune cells, glioblastoma, and the brain. Abbreviations; Short-chain fatty acids (SCFAs), Gut-associated lymphoid tissue (GALT), Blood–brain barrier (BBB), Peroxisome proliferator-activated receptor gamma (PPAR-γ), Adenosine triphosphate (ATP), Regulatory T cell (Treg), Interleukin-10 (IL-10), Transforming growth factor beta (TGF-β), Perforin (Prf), Granzyme B (GzmB), Interferon gamma (IFNγ), and Tumor necrosis factor alpha (TNFα). Created in BioRender. Cox, A. (2024) BioRender.com/z29n756. Available online: https://app.biorender.com/citation/67165a3089a5793f6ce9b641 (accessed on 20 October 2024).

Metabolites, which circulate through the blood and lymphatic vessels and are produced via the gut microbiota, may impact the growth and development of glioma. Metabolites produced via the gut microbiota include tryptophan, arginine, glutamate, glutamine, SCFAs (butyrate, acetate, and propionate), trimethylamine-N-oxide, thiamine, folate, biotin, riboflavin, and pantothenic acid [85] (Figure 2).

Butyrate is one of the main SCFAs, which accounts for ~20% of the total SCFAs present in the gut environment and plays a role as an energy source for the intestinal mucosa and plays a role in cell regulation, proliferation, and differentiation. Most bacteria associated with butyrate production belong to Firmicutes, Actinobacteria, Bacteroidetes, Fusobacteria, and Proteobacteria [47]. Butyrate affects overall innate immunity via the promotion of monocyte differentiation into macrophages. Macrophage phenotypes range from M1 to M2, where M1 are pro-inflammatory and M2 aid in tissue repair and immune suppression [86,87]. Zhou et al. (2024) supported that gut dysbiosis accelerated tumor growth and increased the population of M2 macrophages in the tumor microenvironment (TME), with a reduction in SCFAs. Supplementation of SCFAs reversed this effect by increasing the population of M1-like macrophages and improving glioma outcomes [87]. Butyrate has been shown to suppress genes linked to M2 macrophages, which can result in anti-tumor-like effects, as an increase in M1 macrophages is typically associated with successful cancer treatment [88]. Additionally, butyrate promotes the differentiation of naïve T cells into regulatory T cells (Tregs) and supports the extra-thymic development of Tregs [89]; however, this may impact cancer therapy negatively, as Tregs have been shown to suppress anti-tumor immune response [90]. Butyrate, which is typically synthesized from dietary fiber, has been associated with improved tumor immunotherapy outcomes. It boosts the effectiveness of anti-PD-1 therapy by activating CD8+ T cells [91,92].

SCFAs including propionate have been reported to have protective effects, which include modulation of inflammatory cascade via inhibiting NF-κB and histone deacetylase pathways, as well as immune modulation via interleukins, cytokines, and oxidative stress [49,93]. SCFAs have been suggested as potential anti-cancer agents, particularly in colon and breast cancers. They may promote apoptosis, induce cell detachment, and reduce the overall tumor cell population within the TME. A study by Filippone et al. (2022) revealed that sodium propionate has an anti-tumor-like effect via PPAR-γ/SCFAs signaling, potentially by upregulating PPAR-γ [49]. In GBM, the dysregulated apoptosis enables tumor progression via upregulating angiogenesis, cell migration, and invasive properties. This resulted in a decrease in cell viability, migration, and overall tumor growth of GBM via the promotion of apoptosis and autophagy pathways (mediated via p53 pathways) [49].

Previous data have revealed that acetate can be utilized in GBM or brain metastases [94]. Acetyl-CoA synthetases convert acetate into acetyl-CoA and have been correlated with the development and progression of various cancers, including GBM. When acetate is taken up via cells, only two enzymes have been shown to utilize it as a substrate: acetyl-CoA synthetase 1 (ACSS1), which is in the mitochondria, and acetyl-CoA synthetase 2 (ACSS2), which is found in the nucleocytosol. The regulation of acetate metabolism is increasingly being linked to the expression of ACSS2. This enzyme equips cancer cells with the capacity to efficiently utilize acetate as an energy source. Notably, ACSS2 is found at elevated levels in numerous cancer types and it is typically upregulated in response to hypoxic conditions and nutrient scarcity which means it may play a crucial role in helping cancer cells endure the stresses of the TME [50]. Additionally, Chowdhury et al. (2022) found that CD8+ T cells stimulated by IL-12 resulted in an increase in intracellular acetyl CoA levels and they were able to sustain IFNγ production in a nutrient-depleted, tumor-conditioned media; therefore, CD8+ T cells may require higher concentrations of acetate for optimal function, which potentially result in an anti-tumor effect [95,96,97].

The gut microbiota produces tryptophan and tryptophan metabolites and tryptamine and indolic compounds, which can signal intestinal mucosa, organs, and the brain [98]. A potential route of microbial-derived tryptophan on glioma development is via the aryl hydrocarbon receptor(AHR), a ligand-activated transcription factor that plays a role in cell metabolism, proliferation, differentiation, cell death, and cell adhesion [99]. AHR is also expressed in gliomas; therefore, the gut microbiota is critically involved in dietary tryptophan metabolism and catalyzes tryptophan to produce AHR agonists [100]. Panitz et al. utilized scRNA-seq data and showed that genes associated with tryptophan metabolism were expressed in GBM infiltrating cell types, particularly macrophages and T cells, which exhibited AHR activation. Also, high AHR activity was linked to a reduction in survival in the GBM TCGA dataset [101]. Additionally, the remaining AHR agonists can bind to astrocytes and gliomas which results in T cell activation, the regulation of dendritic cells, and the recruitment of tumor-associated macrophages (TAMs). Furthermore, exogenous consumption of tryptophan resulted in glioma cells activating AHR which inhibited T cell function, induced T cell apoptosis, promoted CD39 expression, and induced differentiation of T cells mediated by interleukin 10 (IL-10) [102]. Lastly, Dono et al. (2020) utilized mass spectrometry and 16S rRNA sequencing on fecal samples, showing that glioma caused significant changes in SCFAs and neurotransmitters, with a reduction in 5-hydroxyindoleacetic acid and norepinephrine. Temozolomide treatment reversed these effects, potentially due to changes in microbial composition, as supported by findings from Patrizz et al. (2020) [23,51].

## 6. Gut Microbiota Signaling Through Pattern Recognition Receptors and Glioma

The gut microbiota also modulates the immune system by activating pattern recognition receptors (PRRs) such as toll-like receptors (TLRs). For example, TLR2 (with either TLR1 or TLR6) is activated by lipoproteins, peptidoglycan, and lipoteichoic acid (LTA) from Gram-positive bacteria, while TLR4/CD14 is activated by lipopolysaccharides (LPSs) from Gram-negative bacteria [103]. These receptors signal through MyD88 and the NF-κB pathway to promote inflammation [104]. While much research has been performed to determine gut microbiota PRR signaling in gastrointestinal (GI) cancers [105,106,107], less is known about its role in other cancer types. However, PRR signaling through endogenous ligands has been more extensively studied in non-GI cancers, and bacterial byproducts have been used as potential therapeutics.

Endogenous TLR2 ligands and the injection of synthetic and bacterial TLR2 ligands tend to be associated with enhanced anti-cancer immune responses [108]. In murine models of UVB-induced skin cancer, LTA administration improved anti-tumor response [109]. In mouse glioma models, high-mobility group box 1 (HMGB1), an alarmin produced by dying cancer cells, activates TLR2 in dendritic cells (DCs) and leads to brain tumor regression [110]. The injection of bacterial lipoprotein improved the anti-tumor efficacy of T cell adoptive transfer therapy in glioma-bearing mice [111]. There is even an ongoing phase I clinical trial using TLR2 ligand Pam3Cys as a treatment for glioma [112].

TLR4 is overexpressed in breast cancer and TLR4 signaling, usually through endogenous ligands from the tumors, improved anti-tumor immunity [113,114,115]. In three different mouse cancers, intratumoral LPS injection increased T cell infiltration, dendritic cell activation, and anti-tumor response [116]. In glioma, TLR4 signaling appears to have a dual role in tumor development and progression. In some mouse glioma studies, LPSs induced tumor cell migration and proliferation, and in others, LPSs increased survival times and immune infiltration into the glioma [117,118]. Despite the strong evidence for the role of TLR2/4 signaling in cancer immune response, the importance of the gut microbiota in modulating the TLR signaling in glioma is largely unexplored.

## 7. Molecular Mimicry Between Microbial and Tumor Antigens in Glioblastoma

Cross-reactivity of T cells allows them to respond to a wider range of antigens. It is most well documented in autoimmune disease where T cells that react to both pathogens and self-antigens lead to the immune attack of healthy human organs. More recently, cross-reactivity between multiple tumor antigens, bacterial antigens, and viral antigens has been documented in various cancers [119,120]. Due to molecular mimicry between gut microbial antigens and tumor antigens, T cell cross-reactivity may be a mechanism for the gut microbiota-driven differences in anti-tumor immune response and immunotherapy efficacy [121]. Computational analysis found significant sequence and structural homologies between tumor-associated antigens and Firmicutes and Bacteriodota antigens [122,123]. Paired tumor and microbial antigens from that study were then tested in T cell cultures from patients with hepatocellular carcinoma, lung cancer, and colon cancer to confirm cross-reactivity and an IFNγ anti-tumor response [124]. In mice with B16 melanomas expressing the SIYRYYG (SIY) neoantigen, the gut commensal *Bifidobacterium breve* was able to stimulate an anti-tumor response through CD8+ T cell receptor (TCR) cross-reactivity between the tumor SIY epitope and the bacterial SVYRYYGL epitope [125]. This has also been shown in GBM, where Naghavian et al. (2023) found tumor antigen-specific T cells from patients with GBM were broadly cross-reactive to multiple bacteria- and gut microbiota-derived peptides [126]. Future therapeutic approaches such as peptide vaccination and fecal microbiota transplantations may be able to use the cross-reactivity between microbial and glioma antigens to improve patient outcomes.

## 8. Conclusions

There is growing evidence that the gut microbiota plays a significant role in cancer development. In glioma, the gut microbiota appears to interact with the tumor through short-chain fatty acids and other metabolites. These interactions can alter immune cell populations, both within the tumor microenvironment and throughout the body, potentially impacting the cancer’s growth and progression. Additionally, differences in microbial species have been associated with the success of cancer treatments, including immunotherapy, suggesting that the composition of the gut microbiota may influence the effectiveness of these therapies. Furthering the understanding of how pattern recognition of the microbiota affects immune modulation and glioma progression will provide insight into underlying mechanisms that may affect overall treatment outcome. Lastly, the molecular mimicry between microbial and tumor antigens highlights a potential novel mechanism of how the microbiota influences immune response in glioblastoma, which may lead to new therapeutic interventions.
